# Revision surgery in anterior cruciate ligament reconstruction: a cohort study of 17,682 patients from the Swedish National Knee Ligament Register

**DOI:** 10.1007/s00167-016-4399-0

**Published:** 2016-12-19

**Authors:** Neel Desai, Daniel Andernord, David Sundemo, Eduard Alentorn-Geli, Volker Musahl, Freddie Fu, Magnus Forssblad, Kristian Samuelsson

**Affiliations:** 1 Department of Orthopedics, Sahlgrenska University Hospital, 431 80 Mölndal, Sweden; 2 Department of Orthopedics, Institute of Clinical Sciences, The Sahlgrenska Academy, University of Gothenburg, Gothenburg, Sweden; 3 Vårdcentralen Gripen, Karlstad, Sweden; 4 Primary Care Research Unit, County Council of Värmland, Karlstad, Sweden; 5 Department of Orthopaedic Surgery, Mayo Clinic, Rochester, MN USA; 6 Department of Orthopedic Surgery, University of Pittsburgh, Pittsburgh, PA USA; 7 Stockholm Sports Trauma Research Center, Karolinska Institutet, Stockholm, Sweden

**Keywords:** Anterior cruciate ligament, Revision, Register, Anatomic, Drilling, Reconstruction

## Abstract

**Purpose:**

To investigate the association between surgical variables and the risk of revision surgery after ACL reconstruction in the Swedish National Knee Ligament Register.

**Methods:**

This cohort study was based on data from the Swedish National Knee Ligament Register. Patients who underwent primary single-bundle ACL reconstruction with hamstring tendon were included. Follow-up started with primary ACL reconstruction and ended with ACL revision surgery or on 31 December, 2014, whichever occurred first. Details on surgical technique were collected using an online questionnaire. All group comparisons were made in relation to an “anatomic” reference group, comprised of essential AARSC items, defined as utilization of accessory medial portal drilling, anatomic tunnel placement, visualization of insertion sites and pertinent landmarks. Study end-point was revision surgery.

**Results:**

A total of 108 surgeons (61.7%) replied to the questionnaire. A total of 17,682 patients were included [*n* = 10,013 males (56.6%) and 7669 females (43.4%)]. The overall revision rate was 3.1%. Older age as well as cartilage injury evident at index surgery was associated with a decreased risk of revision surgery. The group using transtibial drilling and non-anatomic bone tunnel placement was associated with a lower risk of revision surgery [HR 0.694 (95% CI 0.490–0.984); *P* = 0.041] compared with the anatomic reference group. The anatomic reference group showed no difference in risk of revision surgery compared with the transtibial drilling groups with partial anatomic [HR 0.759 (95% CI 0.548–1.051), n.s.] and anatomic tunnel placement [HR 0.944 (95% CI 0.718–1.241), n.s.]. The anatomic reference group showed a decreased risk of revision surgery compared with the transportal drilling group with anatomic placement [HR 1.310 (95% CI 1.047–1.640); *P* = 0.018].

**Conclusion:**

Non-anatomic bone tunnel placement via transtibial drilling resulted in the lowest risk of revision surgery after ACL reconstruction. The risk of revision surgery increased when using transportal drilling. Performing anatomic ACL reconstruction utilizing eight selected essential items from the AARSC lowered the risk of revision surgery associated with transportal drilling and anatomic bone tunnel placement. Detailed knowledge of surgical technique using the AARSC predicts the risk of ACL revision surgery.

**Level of evidence:**

III.

## Introduction

Anterior cruciate ligament (ACL) reconstruction has evolved considerably over the last decades. Surgical factors such as graft selection and fixation methods, drilling techniques and subsequent tunnel placement have been of particular interest. Biomechanical and clinical studies have shown superior results with anatomic reconstruction techniques [7, 11, 12, 20, 31]. In addition, several long-term clinical trials have revealed suboptimal results when non-anatomic techniques are used [4, 5, 13, 21, 25]. Non-anatomic bone tunnel placement is often cited as the most common cause of clinical failure [10, 15, 19, 24]. A multi-centre study by the MARS group revealed that in at least 50% of the revision cases, technical error was either a predominant or contributing factor [28]. Of these, malpositioning of the femoral and/or tibial tunnels were leading causes. Interestingly, several studies have recently shown that anatomically placed grafts are exposed to greater in situ forces than non-anatomically placed ones [3, 11, 16, 29], possibly reflected by results from the Danish Knee Ligament Reconstruction Register showing an increased risk of ACL revision surgery when the transportal (TP) technique was used compared with the transtibial (TT) technique [23]. In contrast, higher odds of repeat ipsilateral knee surgery have been reported in those patients undergoing ACL reconstruction using a TT technique compared with TP technique [8]. The recent introduction of the anatomic anterior cruciate ligament reconstruction scoring checklist (AARSC) provides a tool for evaluating the surgical techniques employed in ACL reconstructions. It has previously been implemented in a systematic review [6]. However, it has yet to be applied to large patient cohorts. The purpose of this population-based cohort study was to analyse data from the Swedish National Knee Ligament Register and apply the AARSC checklist in order to investigate whether detailed knowledge of surgical technique can be used to predict the risk of ACL revision surgery.

## Materials and methods

**Fig. 1:**
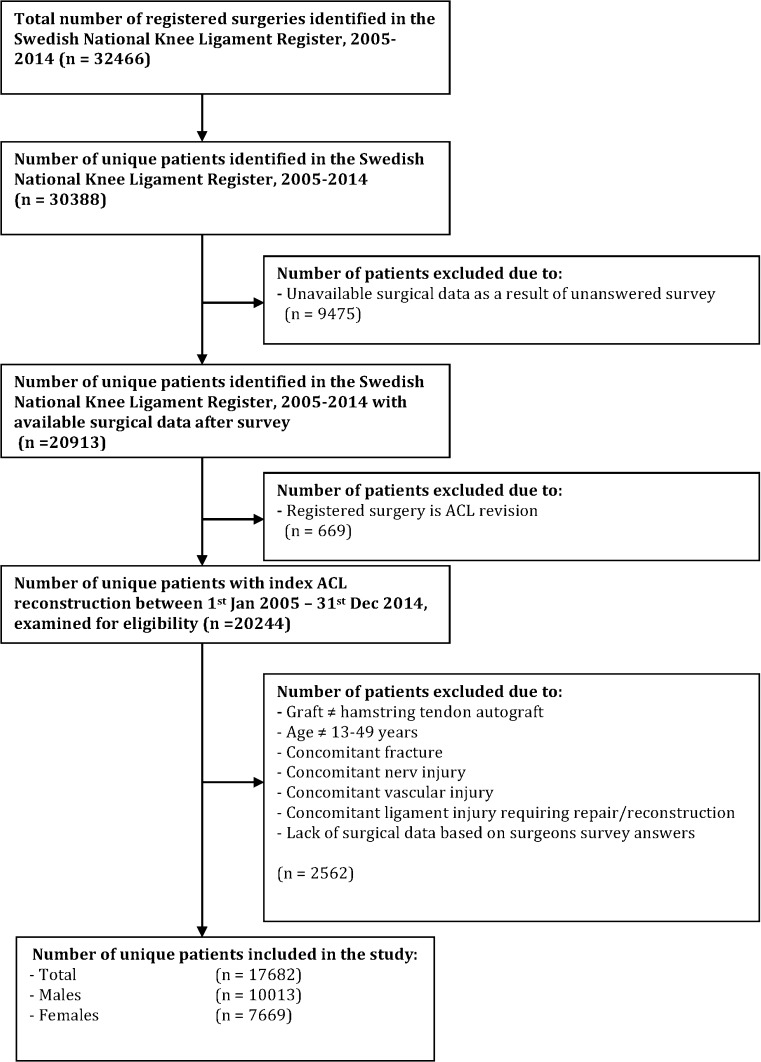
Flow diagram of inclusion and exclusion criteria

Patient data were extracted from the Swedish National Knee Ligament Register. Patients registered for primary ACL reconstruction from 1 January, 2005, to 31 December, 2014 were eligible for inclusion. Only patients aged 13–49 years who underwent primary single-bundle (SB) ACL reconstruction using a hamstrings graft were included. Follow-up started on the date of primary ACL reconstruction and ended with ACL revision surgery, or on 31 December, 2014, whichever occurred first. No minimum follow-up time was pre-specified; instead patients with a possible follow-up shorter than the earliest documented event (revision ACL surgery) in the specific cohort were censored from analysis. Exclusion criteria are summarized in Fig. 1. Data on age at index surgery, patient sex, as well as data regarding graft choice and concomitant injuries noted at index surgery, were extracted from the Swedish National Knee Ligament Register. All data pertaining to surgical technique were gathered via an online questionnaire described below.

An online questionnaire was created to collect detailed information on the surgical technique used by ACL surgeons in Sweden. The questionnaire was based on the items in the anatomic anterior cruciate ligament reconstruction scoring checklist (AARSC) [6, 26]. The checklist allows for calculation of an “anatomic score” with a total of 19 points. The items were translated into Swedish by a professional language editor.

The questionnaire was launched via an online survey (http://lulab.orthop.gu.se/korsbandskirurgi) in January 2015. The 175 surgeons registered in the National Register as of 31 December, 2014 were asked via email to participate. Non-responders were sent three reminders. Data collection ended on 30 April, 2015. For each item in the questionnaire, the surgeon was asked to specify whether they consistently “Always” or “Never” used the surgical technique in question, and whether they still used the technique today. If the surgeon had adapted their surgical technique to subsequently include the item in question, or whether there was uncertainty as to when this change took place, the surgeon had the option to specify by answering the questions: “Never performed until year” and “Always performed after year”, with a specific year chosen from a drop-down menu. This was repeated for items 1–17 in the questionnaire. This resulted in a time interval where it was possible to identify the surgical technique(s) used by each surgeon who responded, as well as the corresponding patients on which these techniques were implemented.

Groups were created using combinations of eight items selected from the AARSC. These eight items were selected by the authors with the aim of forming groups that were considered reflect the surgical techniques utilized during the various stages of evolution of ACL reconstruction seen in recent years. This created comparable groups of adequate size. Each group had a mandatory “Yes” or “No” answer requirement for certain items that subsequently identified that particular group (Table 1). For example, the group “TP-anatomic” was identified by “Yes” answers to “transportal drilling of the femoral ACL tunnel(s)”, “placing the femoral tunnel(s) in the femoral ACL insertion site” and “placing the tibial tunnel(s) in the tibial ACL insertion site”. No pre-specified answer requirements were assigned to the remaining five items for that particular group; to these items surgeons could respond with either “Yes” or “No”.

**Table 1:** Answer requirements characterizing defined groups

	Use of an acc. medial portal	Visualization of the femoral ACL insertion site	Visualization of the tibial ACL insertion site	Lateral intercondylar ridge identified	Bifurcate ridge identified	Placing the femoral tunnel(s) in the femoral ACL insertion site	Placing the tibial tunnel(s) in the tibial ACL insertion site	Transportal drilling of the femoral ACL tunnel(s)
Group								
TP-reference	Yes	Yes	Yes	Yes	Yes	Yes	Yes	Yes
TP-anatomic						Yes	Yes	Yes
TT-anatomic						Yes	Yes	No
TT-partial anatomic						No	Yes	No
TT-non-anatomic						No	No	No
All landmarks		Yes	Yes	Yes	Yes			
No landmarks		No	No	No	No			
TP drilling								Yes
TT-drilling								No

Empty spaces are not assigned a mandatory answer requirement. Surgeons can thus answer “Yes” or “No” to these items

*Acc* accessory, *TP* transportal, *TT* transtibial

All group comparisons were made to a reference group named “TP-reference” (Table 1). The study end-point was ACL revision surgery, defined as replacement of a primary ACL reconstruction. The Regional Ethical Review Board in Gothenburg, Sweden, approved this study (Ref: 760-14).

### Statistical analysis

Tables and diagrams were generated using Microsoft Excel for Mac (Version 14.5.9, Microsoft Corp, Redmond, WA, USA). A statistician assigned to the Swedish National Knee Ligament Register performed all statistical analyses. Statistical analysis was performed in IBM SPSS statistics (Version 23.0, IBM Corp, Armonk, NY, USA). Kaplan–Meier survival analysis was used to assess the cumulative graft survival rates. Statistical significance was defined as a 95% CI for hazard ratios not including 1.0 and a *P* value <0.05. Multivariate analysis adjusted for possible confounding factors (age, patient sex, concomitant injury to menisci or cartilage) was analysed using a Cox regression model and expressed as hazard ratios and 95% confidence intervals (CI). The assumption of proportional hazards was assessed by use of log–log plots.

## Results

**Fig. 2:**
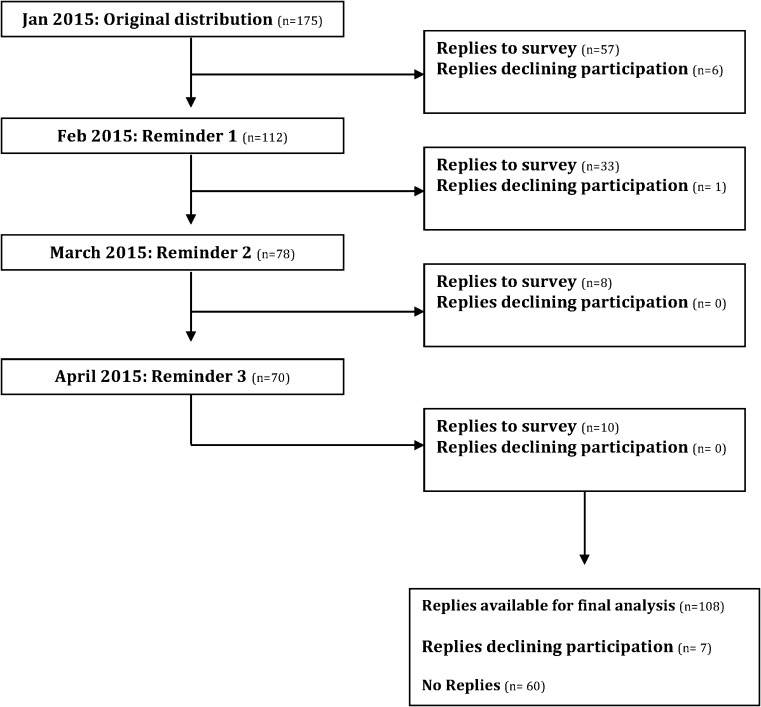
Flow diagram of questionnaire distribution

**Fig. 3:**
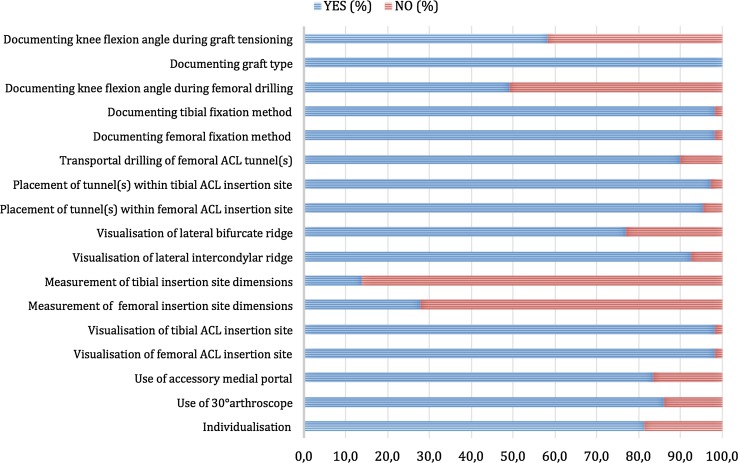
Current national frequency of use of surgical variable amongst questionnaire respondents

**Fig. 4:**
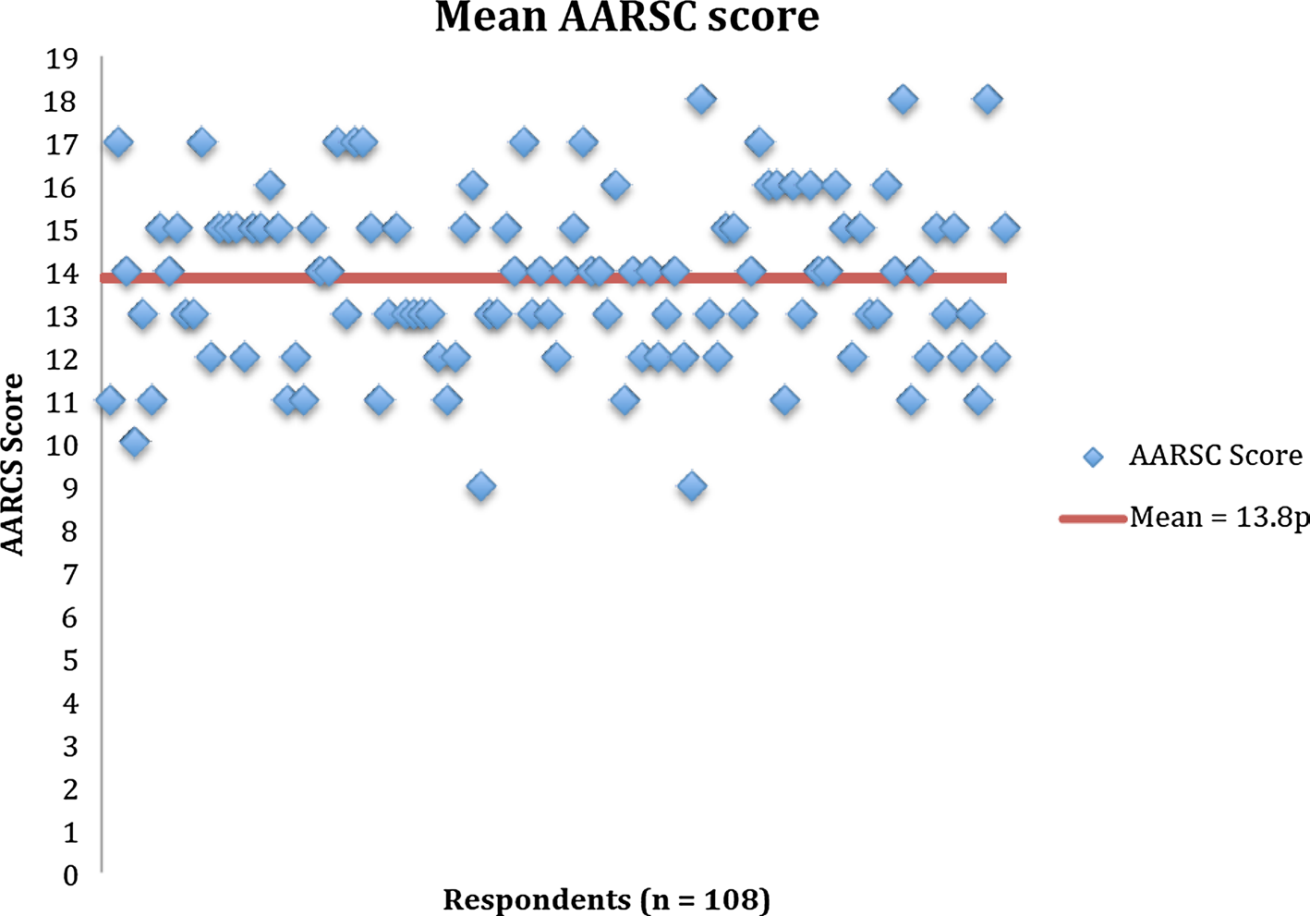
Mean AARSC score based on respondents questionnaire answers

A total of 108 surgeons completed the questionnaire corresponding to a 61.7% response rate. Seven surgeons declined to participate citing inability to accurately complete the questionnaire due to low or absent volume of ACL reconstructions (Fig. 2). Current adoption of the individual items of the AARSC is illustrated in Fig. 3. None of the respondents reported using the highest level of documentation according to the AARSC (yielding 2 points), namely 3D MRI, 3D CT or navigation. The mean nationwide AARSC score based on the questionnaire answers was 13.8 points (Fig. 4).

A total of 17,682 patients were included in the study [*n* = 10,013 males (56.6%) and 7669 females (43.4%)] (Fig. 1; Table 2). The median age at index surgery was 24 years (range 13–49 years). Five patients suffered a contralateral ACL injury and were excluded from the analysis. A total of 552 (3.1%) patients underwent ACL revision surgery [*n* = 296 males (53.6%) and 256 females (46.4%)] (Table 3).

**Table 2:** Description of baseline cohort

	Cohort (*n* = 17,682)	
	%	*N*
Patient sex		
Male	56.6	10,013
Female	43.4	7669
Age at index ACL reconstruction		
13–15 years	7.4	1300
16–20 years	28.7	5075
21–25 years	20.7	3667
26–30 years	14.2	2513
31–35 years	10.0	1777
36–49 years	18.9	3350
Concomitant MCL injury at index surgery		
Yes	2.4	425
No	97.6	17,257
Concomitant LCL injury at index surgery		
Yes	0.6	100
No	99.4	17,582
Meniscus injury present (medial and/or lateral) at index surgery		
Yes	43.8	7743
No	56.2	9939
Cartilage injury present at index surgery		
Yes	26.0	4598
No	74.0	13,084
Meniscus and/or cartilage injury at index surgery		
Yes	54.8	9685
No	45.2	7997

*ACL* anterior cruciate ligament, *LCL* lateral collateral ligament, *MCL* medial collateral ligament

**Table 3:** Patient sex, age and concomitant injury and risk of revision ACL surgery

	Revision cohort (*n* = 552)				
	%	*N*	Hazard rate	95% CI	*P* value
Patient sex					
Male^a^	53.6	296	1.128	0.954–1.333	n.s.
Female	46.4	256			
Age at index ACL reconstruction					
13–15 years	13.4	74	5.259	3.532–7.833	<0.001
16–20 years	45.7	252	4.675	3.297–6.628	<0.001
21–25 years	21.2	117	3.131	2.155–4.548	<0.001
26–30 years	7.8	43	1.590	1.021–2.476	0.040
31–35 years	5.4	30	1.527	0.941–2.479	n.s.
36–49 years^a^	6.5	36			
Meniscus injury present (medial and/or lateral) at index surgery					
Yes	43.1	238	0.994	0.840–1.176	n.s.
No^a^	56.9	314			
Cartilage injury present at index surgery					
Yes	21.2	117	0.720	0.587–0.883	0.002
No^a^	78.8	435			

*ACL* anterior cruciate ligament, *CI* confidence interval

^a^Reference group

### Patient sex

**Fig. 5:**
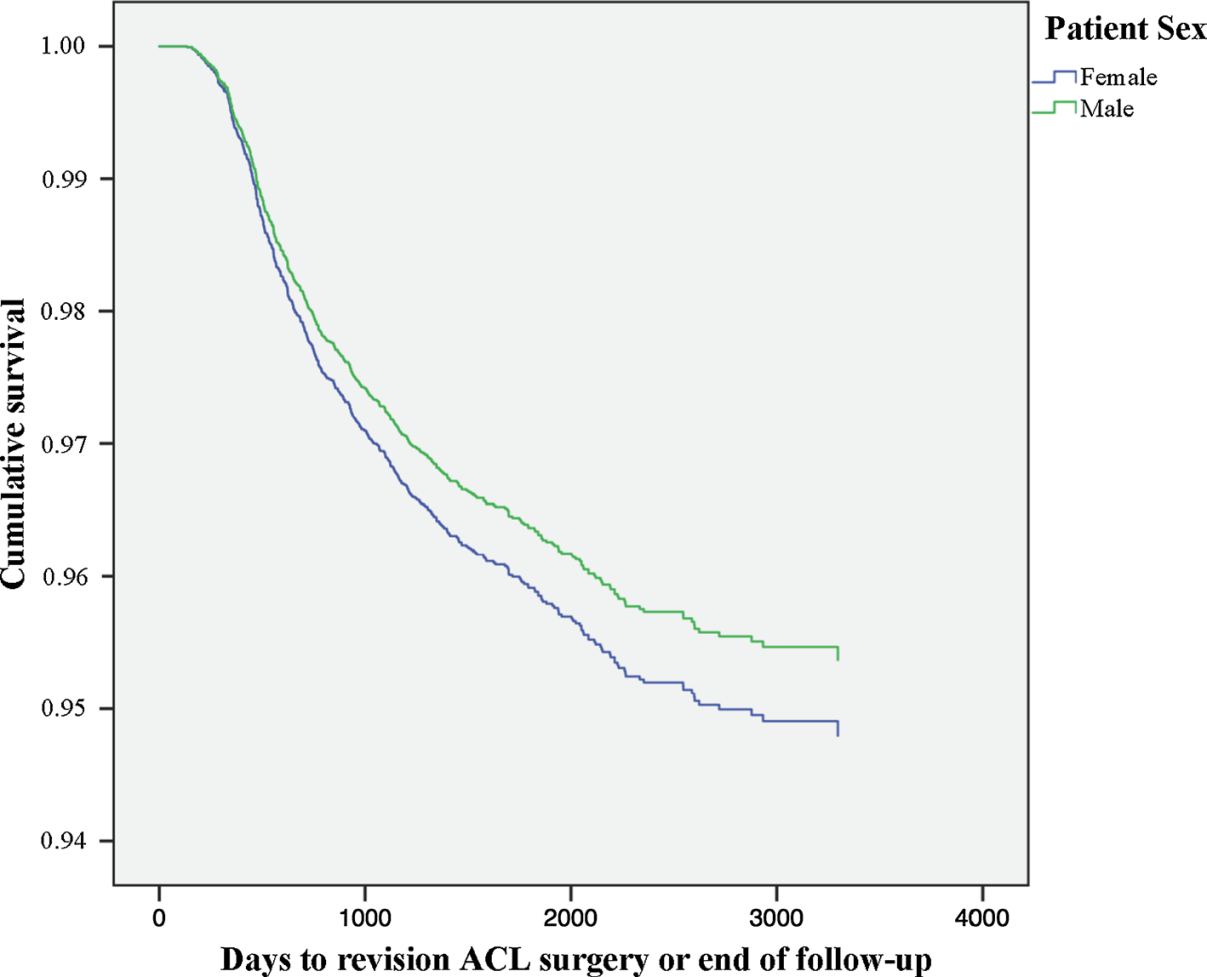
Kaplan–Meier survival function of patient sex and revision ACL surgery

Patient sex was not associated with the risk of revision [HR 1.128 (95% CI 0.954–1.333); n.s.] (Fig. 5; Table 3).

### Patient age

**Fig. 6:**
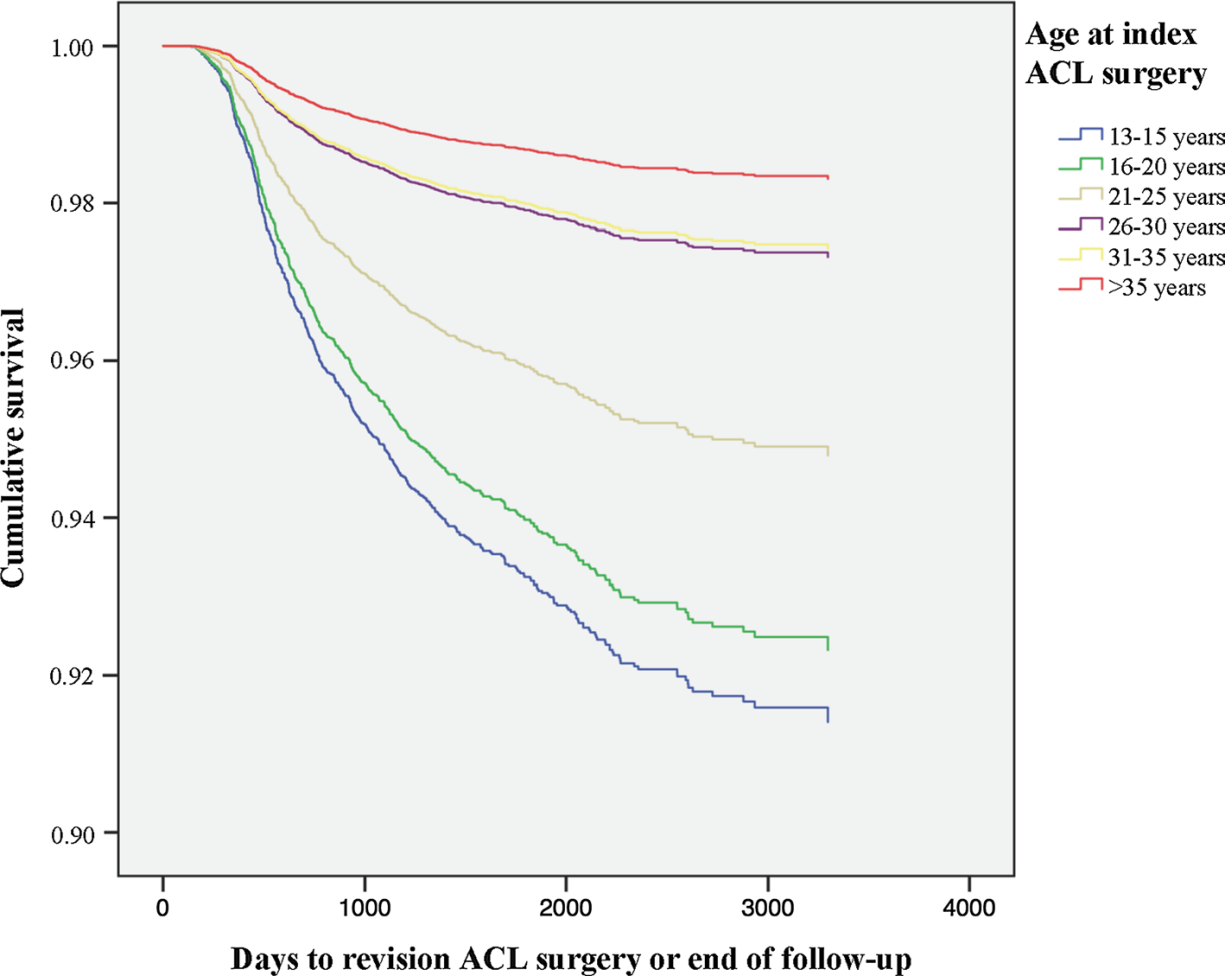
Kaplan–Meier survival function of age at index surgery and revision ACL surgery

The oldest age group (36–49 years) was set as the reference group, and hence, all subsequent comparisons were made to that group (Tables 2, 3). The youngest age group (13–15 years) showed a 5.259-times increased risk of revision compared with the reference age group [HR 5.259 (95% CI 3.532–7.833); *P* < 0.001]. The age group 31–35 years was not associated with the risk of revision compared with the reference group [HR 1.527 (95% CI 0.941–2.479); n.s.] (Fig. 6; Table 3). When stratifying the cohort into two groups and comparing patients 13–25 years of age (*n* = 10,042) with those 26–49 years of age (*n* = 7640), the younger age group showed a 3.19-fold significantly increased risk of revision compared with the older age group [HR 3.19 (95% CI 2.587–3.934); *P* < 0.001].

### Meniscus injury and cartilage injury

**Fig. 7:**
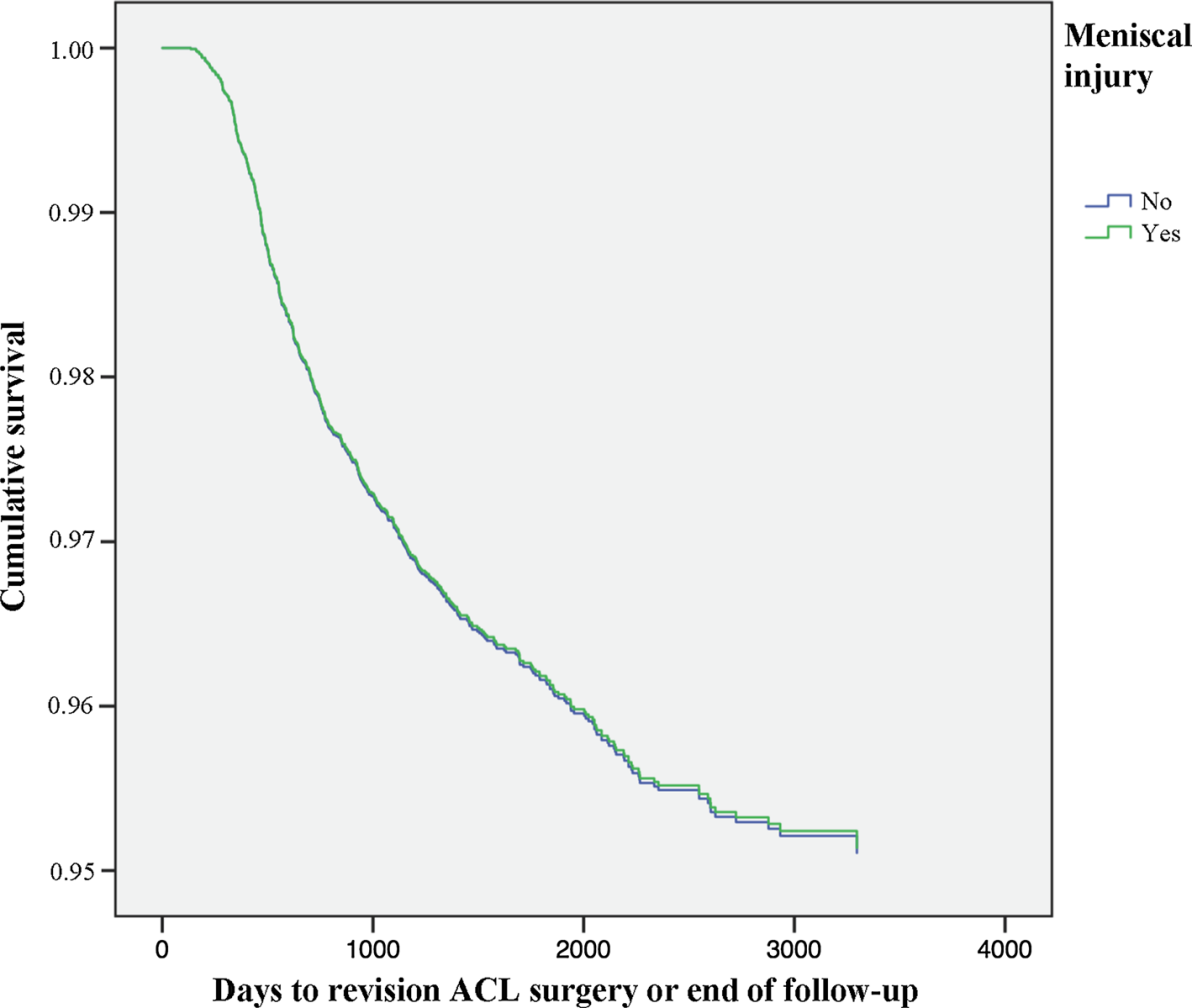
Kaplan–Meier survival function of meniscus injury and revision ACL surgery

**Fig. 8:**
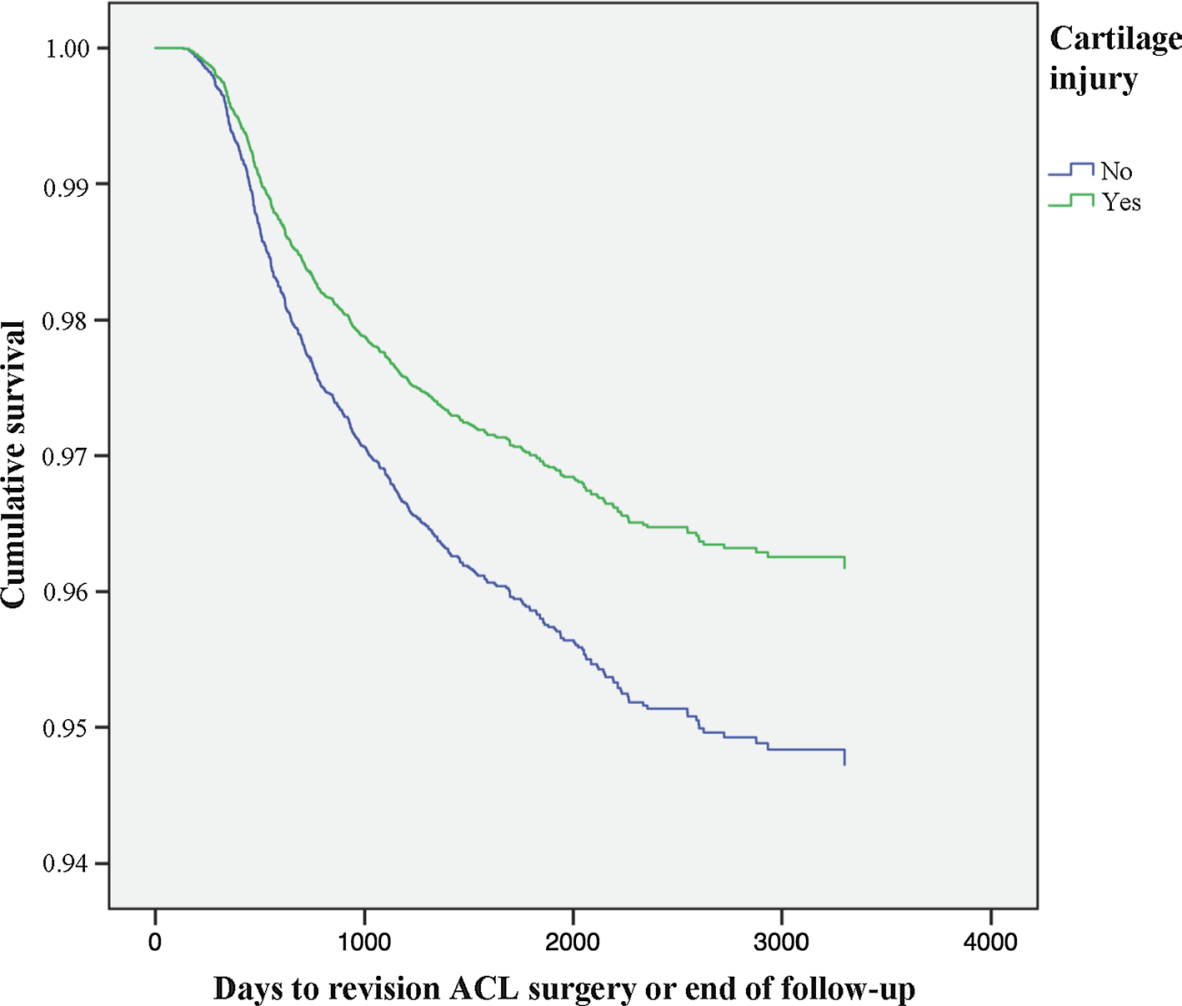
Kaplan–Meier survival function of cartilage injury and revision ACL surgery

Meniscus injury seen at index surgery was not associated with the risk of revision [HR 0.994 (95% CI 0.840–1.176); n.s.] (Table 3). A decreased risk of revision was seen amongst patients with cartilage injury present at index surgery [HR 0.720 (95% CI 0.587–0.883); *P* = 0.002] (Table 3). The combined effect of meniscus and/or cartilage injury observed at index surgery was not an associated with the risk of revision [HR 0.853 (95% CI 0.722–1.088); n.s.] (Table 3; Figs. 7, 8).

### Surgical technique

**Fig. 9:**
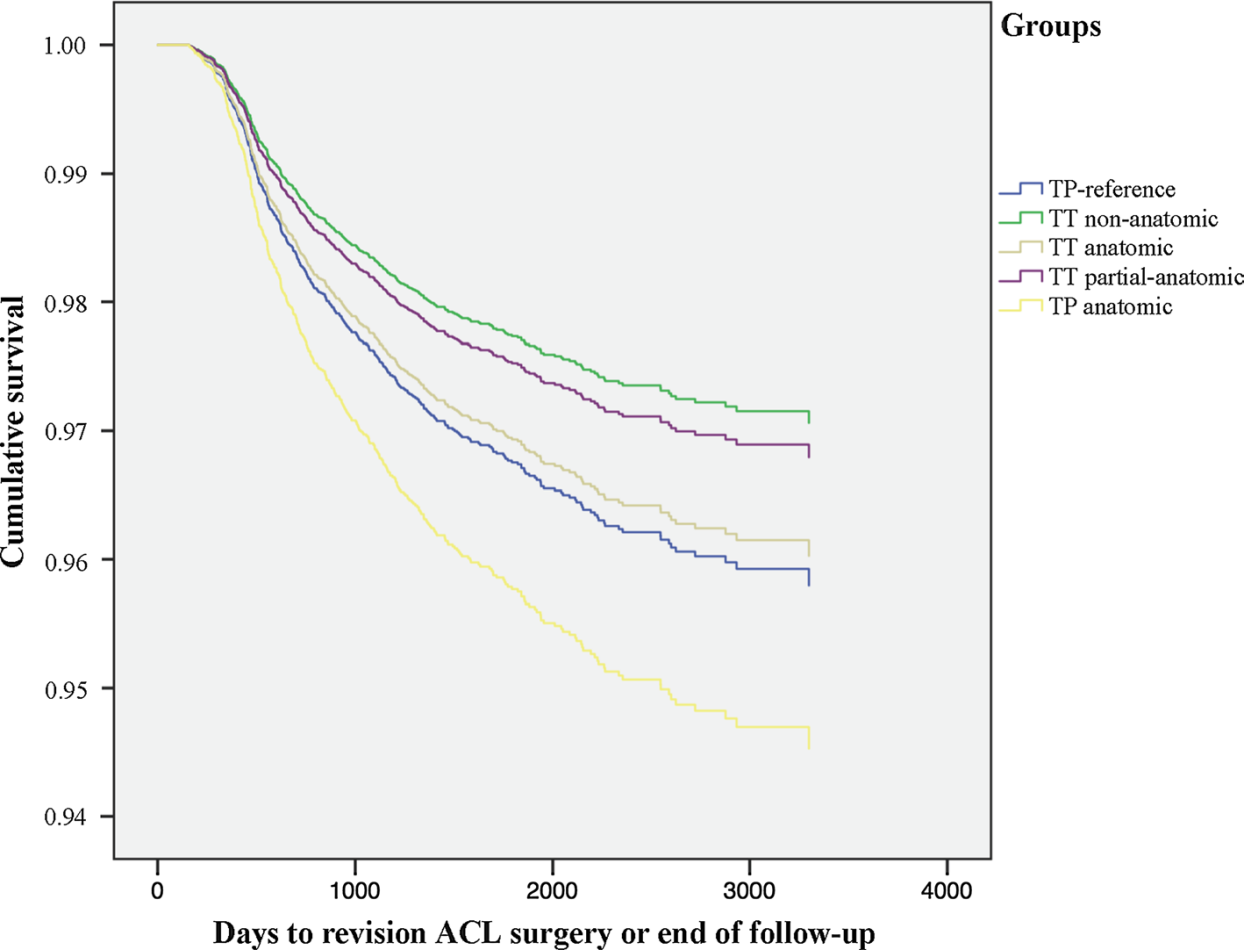
Kaplan–Meier survival function of surgical group and revision ACL surgery

Patients in the TT-non-anatomic group had the lowest risk of revision surgery compared with the TP-reference group [HR 0.694 (95% CI 0.490–0.984); *P* = 0.041]. In contrast, the TP-anatomic group had a higher risk of revision surgery compared with the TP-reference group [HR 1.310 (95% CI 1.047–1.640); *P* = 0.018]. There were no significant differences in risk of revision surgery between the TT-anatomic and TT-partial anatomic groups compared with the TP-reference group (Fig. 9; Table 4).

**Table 4:** Surgical technique and risk of revision ACL surgery

				HR			ADJUSTED HR^a^		
Group				HR	95% CI	*P* value	HR	95% CI	*P* value
Comparison group	No. of events^b^	Reference group	No. of events^b^						
TT-non-anatomic (*n* = 1296)	*n* = 40	TP-reference (*n* = 6685)	*n* = 162	0.704	0.497–0.998	0.049	0.694	0.490–0.984	0.041
TT-anatomic (*n* = 2159)	*n* = 77			0.942	0.717–1.239	n.s.	0.944	0.718–1.241	n.s.
TT-partial anatomic (*n* = 1516)	*n* = 48			0.723	0.522–1.001	n.s.	0.759	0.548–1.051	n.s.
TP-anatomic (*n* = 4036)	*n* = 146			1.285	1.027–1.607	0.028	1.310	1.047–1.640	0.018
All landmarks (*n* = 9398)	*n* = 252	No landmarks (*n* = 831)	*n* = 27	1.387	0.928–2.072	n.s.	1.392	0.931–2.081	n.s.
TP drilling (*n* = 12,440)	*n* = 380	TT-drilling (*n* = 5110)	*n* = 167	1.390	1.157–1.670	<0.001	1.399	1.163–1.682	<0.001

*CI* confidence interval, *HR* hazard ratio, TP transportal, *TT* transtibial

^a^Multivariate Cox regression analysis adjusted for patient sex, patient age and meniscal or chondral injury

^b^Event = revision ACL surgery

### Landmarks

Visualizing all landmarks was not associated with the risk of revision surgery [HR 1.392 (95% CI 0.931–2.081); n.s.] (Table 4).

### Drilling

**Fig. 10:**
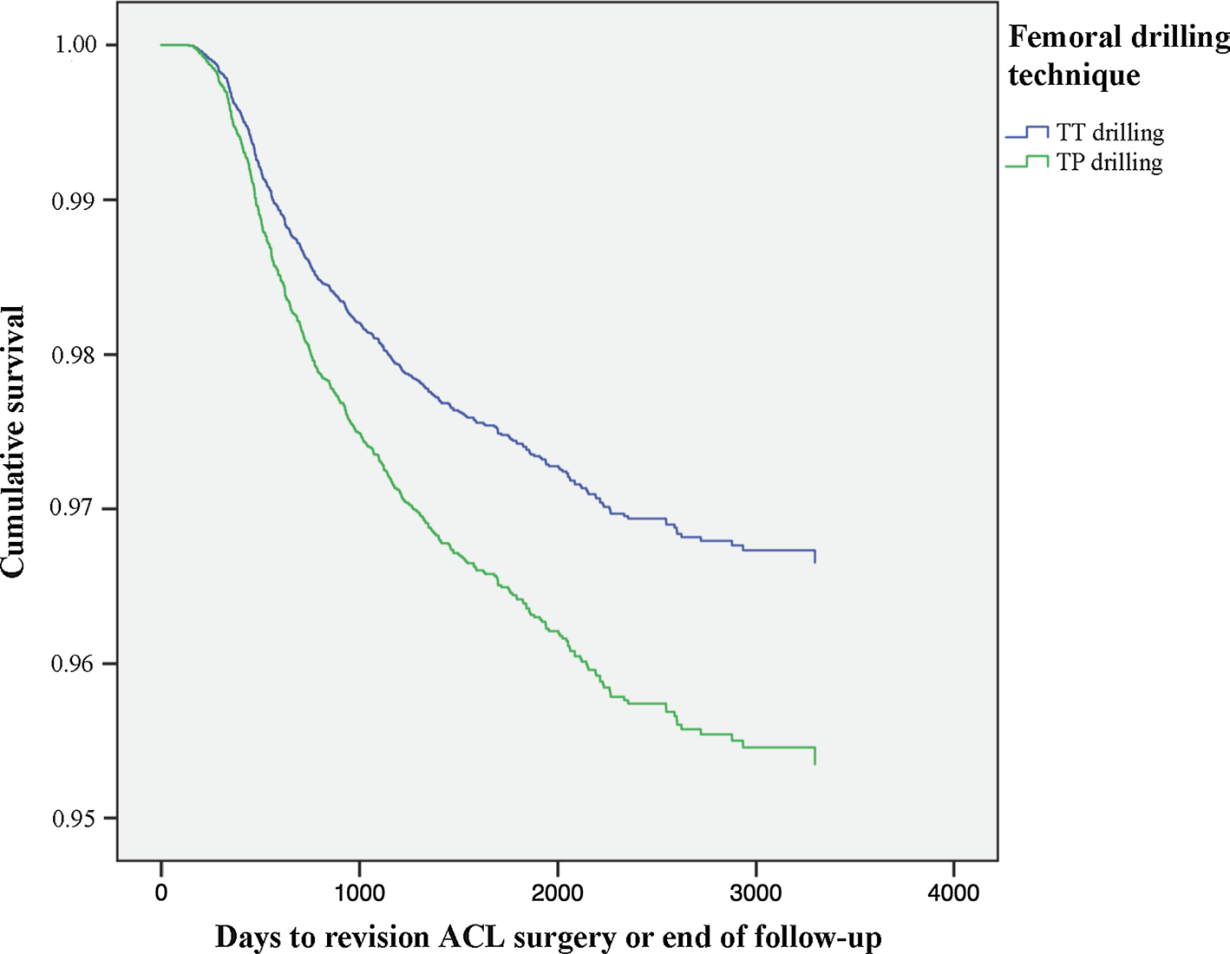
Kaplan–Meier survival function of femoral drilling technique and revision ACL surgery

Transportal femoral bone tunnel drilling was associated with an increased risk of revision surgery compared with transtibial femoral bone tunnel drilling [HR 1.399 (95% CI 1.163–1.682); *P* < 0.001] (Fig. 10; Table 4).

## Discussion

The most important finding of the present study was that the anatomic reference group, comprised of eight essential AARSC items, had a lower risk of revision surgery compared with anatomic bone tunnel placement via transportal drilling. Non-anatomic bone tunnel placement via transtibial drilling resulted in lowest risk of revision surgery. The mean AARSC score based on the respondent’s answers was 13.84, which reflects a promising trend that surgeons are adopting more anatomic techniques.

### Surgical technique

#### Groups

The patients were divided into separate groups based on the surgical techniques adopted by the orthopaedic surgeon. This created a unique possibility to compare different surgical techniques with each other. Patients in the TT-non-anatomic group, with mandatory placement of the graft outside the femoral and tibial footprints, had the lowest risk of revision compared with all other groups. Since these grafts are placed non-anatomically, the forces applied to the grafts are possibly lower [3, 11, 16, 29]. Furthermore, incorrect placement of a graft will likely result in a residual rotational laxity of the knee, thus creating persisting instability [7, 20]. Such instability may lead to adaptation of patient behaviour and activity level, decreasing the risk of a re-rupture of the graft. In addition to this, the residual laxity may lead to increased osteoarthritic changes that in turn, with time, stabilize the knee, reducing the need for revision surgery.

A unique finding in this study was that the reference group with a more complete anatomic reconstruction technique, with the visualization of both footprints, identification of the ridges and anatomic tunnel placement via transportal drilling utilizing an accessory medial portal, showed a decreased risk of revision compared with transportal drilling and anatomic tunnel placement on the femur and tibia. Surgeons performing reconstructions according to the TP-reference group may be more experienced, performing larger volumes of reconstructions per year, possibly explaining the difference in risk of revision surgery. Furthermore, the “TP-reference” group produced similar revision risks compared with patients who underwent reconstruction with transtibial bone tunnel drilling, regardless of the actual graft placement on the femur (TT-partial anatomic). This is also a novel finding revealing that anatomic reconstruction is not inferior to TT femoral drilling techniques. Altogether, this study shows that simply grouping techniques into transtibial and transportal drilling, without further surgical data is not enough and clearly creates a confounding effect that is not adjusted for.

Interestingly, patients in the TP-anatomic group had the highest risk of revision surgery compared with all other groups. An inherent learning curve as well as increased graft forces are two of many factors that have been proposed as reasons for the increased revision frequency. Both seem logical and anatomic placement of the graft has shown less residual laxity which in turn correlates to increased forces on the graft [3, 11, 16, 29]. Studies have yet to show the effects of a learning curve in anatomic reconstruction.

Its is noteworthy that looking solely at crude revisions rates between the surgical groups and drilling techniques, in certain cases revealed results in contradiction to the adjusted hazard rates. This, however, can be explained by the difference in detection times between the two groups being compared, and this is subsequently accounted for during the Cox regression analysis.

#### Drilling

Femoral tunnel drilling through an accessory portal was associated with an increase in revision surgery. Rahr-Wagner et al. [23] have previously reported similar findings. However, when more surgical factors are accounted for, this study shows that the drilling technique is a confounding factor. First, patients in the TT-non-anatomic group had the lowest risk of revision. These patients would also be categorized only according to their drilling technique and potentially skewing the results. Second, the TP-reference group revealed similar results compared with the TT-partial-anatomic and TT-anatomic groups. This suggests that modern anatomic ACL surgery produces equivalent results compared with modern transtibial techniques. A scientific comparison should therefore always include detailed surgical data, preferably items from the AARSC checklist, as these might act as important confounding factors. Finally, these results may indicate a learning curve is inherent to anatomic reconstruction entailing that reconstruction techniques used during the dawn of anatomic ACL reconstruction are possibly not equivalent to today’s modern TP-reference group, making comparisons between patients operated during these separate time periods difficult.

#### Patient sex

Secondary findings of this study found that patient sex did not influence risk of revision. These findings are similar to current literature on the subject [1].

#### Patient age

Younger age was associated with an increased risk of revision with a more than fivefold increased risk of revision in the group 13–25 years and more than fourfold increased risk of revision in the group 16–20 years compared with older patients (36–49 years). This may be a result of younger patients having a higher activity level both pre-injury and subsequently post-operatively, therefore, exposing the graft to deleterious loads. It may also be a consequence of a lack of compliance to post-operative rehabilitation regimes and restrictions, resulting from an over-eagerness to return to activity. It is also possible that younger patients have higher demands and expectations on the reconstruction and opt for revision surgery to a greater extent. There is a possibility that other biological factors play a part too; however, this is not confirmed in current literature. The association between younger patient age and increased risk of graft failure and revision surgery is well established in current literature [2, 9, 14, 17, 18, 22, 27, 30].

#### Meniscal and cartilage injuries

Cartilage injury at the time of index reconstruction was a factor associated with decreased risk of revision surgery. Meniscal injuries were not found to influence risk of revision surgery. Although not fully understood, several reasons have been proposed [1]. Cartilage and meniscal injury may be indicative of a significant initial knee trauma, possibly entailing a reduction in activity level in those patients post-operatively. In addition, the presence of concomitant injuries may entail extended rehabilitation protocols, both pre- and post-operatively, which possibly could possibly be beneficial in terms of graft failure and revision. A consequence of this could also be a prolonged time between injury and surgery, potentially shifting an eventual revision outside of the scope of this study’s follow-up.

## Limitations

An important limitation is that the primary end-point was revision surgery, which fails to identify the total number of graft failures, as not all failures opt to undergo revision surgery. In addition, information on activity level is not available in the register. In this study, a retrospective analysis was performed through an online questionnaire on surgical data, which in turn can entail an element of a recall bias. Assuming honest answers, the surgeon can still erroneously recall dates when a certain technique was adopted. To minimize recall bias, responders were informed to only answer the question whether they were sure of the date they adopted or abandoned the surgical technique in question. In addition, responders were asked to only specify years and not months in an attempt to further minimize recall errors. Moreover, all patients that were operated on during time periods when the surgeon was “in-between” surgical techniques were not included. No verification of the surgical techniques utilized by the non-responders to the questionnaire was undertaken, creating risk of selection bias. The results of this study are only applicable to ACL reconstructions using hamstring grafts. The cause of failure and subsequent revision after ACL surgery and after ACL reconstruction is not solely influenced by the surgical technique used. The present study has adjusted analysis for some of these possible influential factors, but not all of them, and conclusions must be made with this in mind.

## Conclusion

Overall revision rate was low. Non-anatomic bone tunnel placement via transtibial drilling resulted in the lowest risk of revision surgery after ACL reconstruction. The risk of revision surgery increased when using transportal drilling. Performing anatomic ACL reconstruction utilizing eight selected essential items from the AARSC lowered the risk of revision surgery associated with transportal drilling and anatomic bone tunnel placement. Detailed knowledge of surgical technique using the AARSC predicts the risk of ACL revision surgery.
